# Nucleic Acid Thermodynamics Derived from Mechanical Unzipping Experiments

**DOI:** 10.3390/life12071089

**Published:** 2022-07-20

**Authors:** Paolo Rissone, Felix Ritort

**Affiliations:** Small Biosystems Lab, Condensed Matter Physics Department, University of Barcelona, Carrer de Martí i Franqués 1, 08028 Barcelona, Spain; rissone.paolo@gmail.com

**Keywords:** single-molecule biophysics, nucleic acid thermodynamics, statistical mechanics, fluctuation theorems, computational biophysics

## Abstract

Force-spectroscopy techniques have led to significant progress in studying the physicochemical properties of biomolecules that are not accessible in bulk assays. The application of piconewton forces with laser optical tweezers to single nucleic acids has permitted the characterization of molecular thermodynamics and kinetics with unprecedented accuracy. Some examples are the hybridization reaction between complementary strands in DNA and the folding of secondary, tertiary, and other heterogeneous structures, such as intermediate and misfolded states in RNA. Here we review the results obtained in our lab on deriving the nearest-neighbor free energy parameters in DNA and RNA duplexes from mechanical unzipping experiments. Remarkable nonequilibrium effects are also observed, such as the large irreversibility of RNA unzipping and the formation of non-specific secondary structures in single-stranded DNA. These features originate from forming stem-loop structures along the single strands of the nucleic acid. The recently introduced barrier energy landscape model quantifies kinetic trapping effects due to stem-loops being applicable to both RNA and DNA. The barrier energy landscape model contains the essential features to explain the many behaviors observed in heterogeneous nucleic-acid folding.

## 1. Introduction

In molecular biophysics, the accurate knowledge of the thermodynamics of nucleic acids (NAs) and proteins is essential to obtain reliable predictions of protein folding [1,2,3,4,5], DNA/RNA hybridization [6], and their interactions with enzymes and ions [7]. Over the past decades, measurements of molecular free-energies, entropies, and enthalpies have been obtained by using bulk techniques such as UV absorbance, fluorescence, and calorimetry, among others [8]. DNA and RNA hybridization are key reactions in many biochemical processes, such as NA synthesis, RNA folding, and DNA amplification by PCR. The energy parameters used to model the hybridization reaction can be directly obtained from melting curves of oligos of varying sequence and length. Accurate knowledge of these parameters is crucial in many ways, such as optimizing heating–cooling protocols for PCR products, structural predictions in NAs folding and biosensor devices that use DNA hybridization for detection (e.g., surface-plasmon resonance), and sequencing technologies. Unified sets of energy parameters have been derived from DNA and RNA melting temperature data obtained by many laboratories worldwide [9,10]. They are currently used as reference values by all main prediction tools [11].

**Figure 1:**
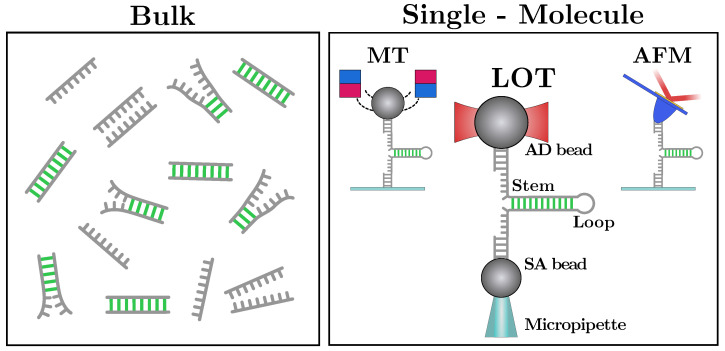
Information about biomolecules from bulk essays (**left panel**) is not a direct procedure and is obtained from the global behavior of a large number of molecules. On the contrary, single-molecule techniques (**right panel**) such as atomic-force microscopy (AFM), magnetic tweezers (MT), and laser optical tweezers (LOT), allow us to sample reactions one molecule at a time, characterizing the molecules at the microscopic level.

However, bulk methods yield results that are incoherent temporal averages over a large population of molecules that are in different states (Figure 1, left). The measured signal depends on the dominant species and reactions, limiting the capability of detecting fast events, rare non-native states, and reaction pathways [12]. For example, RNAs and proteins often become trapped in non-productive, misfolded structures [3,13,14,15,16,17]. Such structures have been related to the development of many phenotype diseases such as Huntington’s disease, fragile X-associated tremor ataxia syndrome, myotonic dystrophies, and spinocerebellar ataxias, among others [18,19,20]. By monitoring one molecule at a time, single-molecule techniques allow for the characterization of these structures, which go undetected in bulk assays. Notice that by averaging results over many events, single-molecule measurements approach the bulk limit to which results can be compared.

By overcoming the intrinsic limitations of bulk measurements, techniques such as single-molecule fluorescence [21], single-molecule translocation across nanopores [22], atomic-force microscopy (AFM), magnetic tweezers (MT), and laser optical tweezers (LOT) [23] offer a fresh view in biomolecular sciences (Figure 1, right panel). In particular, LOTs [24] were revealed to be a powerful tool to investigate the properties of NAs. In fact, LOTs permit the direct measurement of the mechanical work (and therefore of the free-energy) needed to unfold DNA and RNA hairpins, rendering force spectroscopy a valuable tool for NA thermodynamics [25,26]. Moreover, single-molecule manipulation sets a new bar for resolving complex molecular reactions [27], such as NAs’ elastic response [28,29] and non-specific secondary structure formation [30], as well as their protein and NA folding [31,32].

During the past decade, single-molecule techniques have become standard for investigating NAs at the structural, biochemical, and thermodynamical levels. Here, we briefly review the thermodynamics of DNA and RNA folding as obtained by developments in our lab over the past ten years that combine LOTs with suitable data analysis methods. In this way, it has been possible to measure DNA and RNA base-pair free-energies from mechanical unzipping experiments with high accuracy (0.1 kcal/mol) in different experimental conditions [33,34,35]. Unzipping consists of pulling apart the $3^{\prime }$ and $5^{\prime }$ ends on one side of a helical NA structure stabilized by complementary (Watson–Crick) base-pair interactions. The reverse process, called rezipping, consists of the reformation or assembly of the helical structure (hybridization) by approaching the $3^{\prime }$ and $5^{\prime }$ ends. Even though both DNA and RNA form double-stranded helices, important differences are found in unzipping experiments. In particular, unzipping is a fully reversible process for DNA in a broad range of salt conditions and loading rates. Instead, RNA unzipping turns out to be strongly irreversible in the same experimental conditions. In this case, transient off-pathway misfolded structures appear during the unzipping–rezipping process, slowing down the hybridization reaction [36,37,38]. The characterization of these off-pathway structures that compete with the native stem is a challenging problem that requires the extraction of the free energies of kinetic (non-native) states from irreversible work measurements [25,39,40]. In unzipping experiments, DNA and RNA molecules unravel into a single NA chain (single-strand) of a given molecular extension. In contrast, in bulk experiments, no forces are involved, and the unfolded state is a relaxed random coil. To relate unzipping experiments with bulk measurements, it is necessary to correct free energy differences by the so-called stretching energy contribution. This term equals the work needed to stretch the NA chain from the random coil state (zero extension) to the elongated state (finite extension). Therefore, thermodynamic measurements with force spectroscopy require accurate knowledge of the ideal elastic response of the NA chains, which can also be measured by pulling the individual single-strands [29,30]. Here, we discuss the experimental results obtained in our lab and interpret them in the framework of the barrier energy landscape (BEL) model recently introduced by us to explain the strong irreversibility observed in RNA unzipping experiments [35]. We also extend the BEL model to predict of the force–extension curves reported in studies of the non-specific secondary structure in single-stranded DNA [30].

The paper is organized as follows: In Section 2, we describe the LOT setup and the experimental protocol to unzip NAs. In Section 3, we introduce the popular nearest-neighbor model used to characterize the hybridization reaction in DNA and RNA duplexes. In Section 4 we review the results obtained by unzipping long DNA and RNA hairpins with LOTs. In Section 5, we introduce the BEL model, which can be used to explain the strong irreversibility observed in RNA unzipping and the formation of non-specific secondary structures in DNA [30]. Finally, in Section 6, we discuss future perspectives.

## 2. Mechanical Unzipping of Nucleic Acids (NAs)

**Figure 2:**
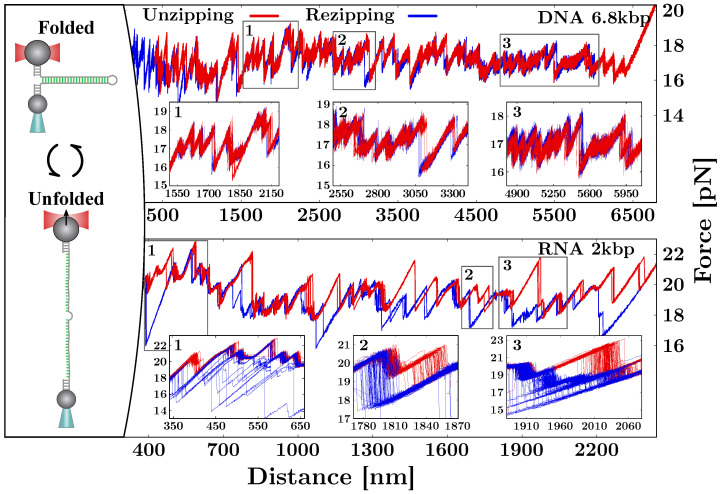
Unzipping experiment with LOTs. (**Left**) In a pulling experiment, the molecular construct is repeatedly unzipped and rezipped. By starting in the folded configuration (**top**), the trap–pipette distance is increased until the molecule is fully unfolded (**bottom**). Then, the process is reversed, and the hairpin refolds upon moving closer the optical trap to the micropipette. (**Right**) The typical FDCs obtained for a 6.8 kbp DNA hairpin (**top**) and a 2 kbp RNA hairpin (**bottom**) in a 1 M NaCl buffer and a 500 mM NaCl buffer, respectively. The insets magnify selected regions (gray squares) along the FDCs to point out the large irreversibility observed in RNA. In contrast, DNA unzipping is fully reversible.

To measure the NAs free-energy of formation at the single base-pair (bp) level, we unzip long DNA and RNA hairpins (a few thousand bases) consisting of a stem of fully complementary Watson–Crick base-pairs (Figure 2). The stem terminates in a tetra-loop GAAA. When fully unzipped, the hairpin converts to the single-stranded form. On the other side, the $3^{\prime }$ and $5^{\prime }$ ends of the hairpin are ligated to short (29 bp) double-stranded handles, one labeled with a digoxigenin tail (DIG) and the other with biotin (BIO). The two tags specifically bind to anti-DIG (AD)- and streptavidin-coated (SA) beads, respectively. The AD bead is optically trapped, while the SA one is held by air suction at the tip of a glass micro-pipette (Figure 1, right panel). In an unzipping experiment, the optical trap is moved with respect to the (fixed) micro-pipette at a constant speed. At the beginning of the protocol, the molecule is folded into its native double-stranded (ds) hairpin configuration (Figure 2, top-left). As the optical trap moves away from the pipette, the force applied to the hairpin increases until the intramolecular bonds at the beginning of the stem break open. As the unzipping progresses, groups of new bases open one after another in a sequential fashion. Unzipping is a stick–slip process consisting of the succession of an elastic deformation (stick) followed by the release of groups of bases that collectively unfold in a cooperative manner (slip), resulting in sudden force jumps. The unfolding protocol proceeds until the hairpin is fully unzipped and the single-strand (ss) form is fully stretched (Figure 2, bottom-left). At this point, the reverse process starts (rezipping), and the molecule refolds starting from the loop until the native ds hairpin has been reformed. Upon rezipping, groups of bases are cooperatively absorbed into the stem resulting in sudden increases in force. The force–distance curve (FDC) measured during unzipping and rezipping exhibits a saw-tooth pattern that depends on the sequence of the hairpin. In Figure 2 (right), we show the FDCs obtained by unzipping a 6.8 kbp DNA hairpin (top) in a 1 M NaCl solution and a 2 kbp RNA hairpin in a 500 mM NaCl solution (bottom). Notice that RNA FDCs exhibit many irreversible regions (insets) with large hysteresis and many long-lived intermediates. This does not happen in DNA, where unzipping experiments are carried out at equilibrium.

## 3. Modeling the Unzipping Experiment

Single-molecule experiments can be used to measure the hybridization free-energy of NA, the interaction energy between hairpins and proteins [41,42,43], and ions [44]. They can also be used to monitor the folding pathway and detect intermediates and misfolded states [40]. As explained in the introduction, force spectroscopy measurements require the knowledge of the contributions of the experimental apparatus (optical trap) and the stretched molecular construct (handles and single-stranded NA). In fact, the standard free-energy of formation of a hairpin, $\Delta G_{0}$, is defined as the free-energy difference between the native and the random coil state, the latter being the state (with zero extension) of the unfolded semi-flexible single-stranded NA when no external force is applied. However, in mechanical unzipping experiments, the unfolded molecule does not attain a random coil but a stretched semiflexible form, making the free energy difference $\Delta G_{\mathrm{tot}}\neq \Delta G_{0}$. To obtain $\Delta G_{0}$, we need to subtract the contribution by the experimental setup to the measured $\Delta G_{\mathrm{tot}}$. Therefore, a theoretical understanding of the experimental contributions to $\Delta G_{\mathrm{tot}}$ is essential to derive reliable free energy values.

### 3.1. Bases Hybridization and Nearest-Neighbors Model

**Figure 3:**
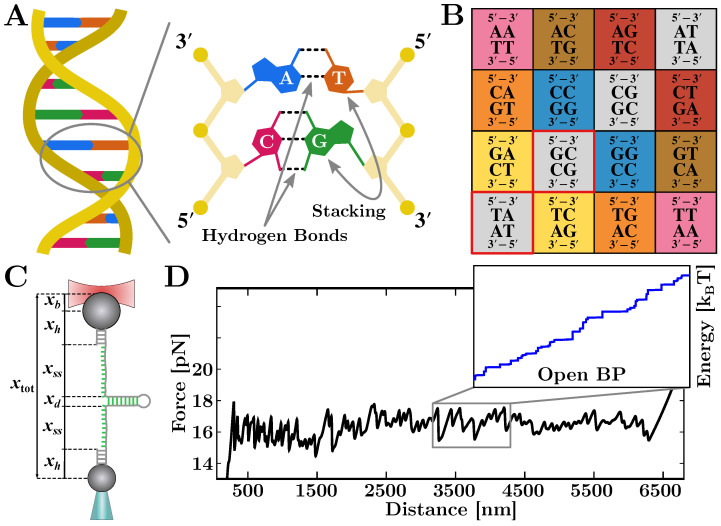
(**A**) Double-helix structure according the Watson–Crick base-pairing rules. (**B**) Matrix of the 16 NNBP motifs according to the NN model. Degenerate energies have the same cell color. Out of the 10 independent parameters, circular symmetry allows to express 2 NNBP energies (TA/AT and GC/CG—red-bordered cells) as a linear combination of the others (see Equation (2)). Notice that the same matrix is obtained for the RNA case by changing thymine (T) for uracil (U). (**C**) Different contributions of the molecular construct to the total extension. Each term (optical trap, dsDNA handles, ssDNA, and folded double-helix) has a different elastic response to the applied force. (**D**) Theoretical prediction of the FDC and of the minimum free-energy as a function of the open bp (inset).

NAs are polymeric chains of monomers, the nucleotides that are linked together by covalent and non-covalent interactions [45] (see Figure 3A, left). The nucleotides are divided into purines, i.e., Adenine (A) and Guanine (G), and pyrimidines, i.e., Cytosine (C) and Thymine (T). For RNA, the nucleotide Uracil (U) substitutes Thymine. A nucleotide (see Figure 3A, right) is formed by one molecule of phosphoric acid (dark-yellow circle), one molecule of $2^{\prime }$-deoxyribose (light-yellow pentagon), and a nitrogenous base (which can be A,C,G,T/U). Two nucleotides concatenate through a bond between the phosphoric group of the first nucleotide and the third carbon of the second nucleotide. The concatenation of multiple nucleotides forms a phosphate–deoxyribose backbone of linked bases featuring a phosphate group on one terminus ($5^{\prime }$) and a hydroxyl group on the other one ($3^{\prime }$). The structure of NAs is conventionally given as a sequence of bases in the direction $5^{\prime }\to 3^{\prime }$.

The canonical interactions between nucleotides belonging to different strands are given by the Watson–Crick base-pairing rules [46] and account for purine–pyrimidine bonding: A links to T (U in the RNA case) and G to C with three and two hydrogen bonds, respectively. Although hydrogen bonding is responsible for the specificity of base interactions, much of the stability of the NA is due to base stacking [47]. In fact, nitrogenous planar bases are non-polar, and the hydrophobic stacking on top of each other maximizes the Van der Waals attraction. To account not only for the specific base-pairing but also for the stacking between adjacent base-pairs, the duplex energetics is described with the nearest-neighbor (NN) model [9,48,49,50].

In the NN model, the base-pairing energy of two complementary bases only depends on the base itself and on the first neighbor located in the same strand (in the $5^{\prime }\to 3^{\prime }$ direction). Therefore, the total free energy of formation of a duplex, $\Delta G_{0}$, is given by the sum over all the nearest-neighbor base-pair (NNBP) motifs occurring along the sequence:

(1) $$\Delta G_{0}\left(N\right)=\sum_{i=1}^{N}\Delta g_{i}\;$$

where $\Delta g_{i}$ is the free energy of motif *i*. Notice that the NNBP energies are negative, as they are defined as the free-energy loss upon hybridizing a base-pair, i.e., $\Delta g_{i}=g_{i}^{H}-g_{i}^{O}<0$, where $g_{i}^{H}$($g_{i}^{O}$) is the free-energy of the hybridized (open) motif. There are 16 different motifs accounting for all possible combinations of adjacent NNBPs (see Figure 3B). This number is reduced from 16 to 10 by considering the degeneracy of the free-energies due to the Watson–Crick complementarity. It is possible to further reduce this number from 10 to 8 independent parameters by considering the circular symmetry of the NN model [51,52]. This symmetry yields additional self-consistent relations so that out of the 10 NNBP energies, 2 can be expressed as linear combinations of the remaining 8 [34,52,53]. Motifs TA/AT (UA/AU) and GC/CG are usually expressed as a function of the others:

(2) $$\begin{array}{cc} \Delta g_{\mathrm{TA}(\mathrm{UA})} & =\Delta g_{\mathrm{CG}}+\frac{1}{2}\left(\Delta g_{\mathrm{AC}}+\Delta g_{\mathrm{GA}}-\Delta g_{\mathrm{AG}}-\Delta g_{\mathrm{CA}}\right)\\ \Delta g_{\mathrm{GC}} & =\Delta g_{\mathrm{AT}(\mathrm{AU})}+\frac{1}{2}\left(\Delta g_{\mathrm{GA}}+\Delta g_{\mathrm{CA}}-\Delta g_{\mathrm{AG}}-\Delta g_{\mathrm{AC}}\right) \end{array}$$

where $\Delta g_{\mathrm{XY}}$ indicates the free energy of motif 5${}^{\prime }$-XY-3${}^{\prime }$ hybridized with its complementary sequence. The accurate measure of the NNBP free-energies is key for the correct estimation of the total free energy of formation of the duplex native state. The 10 independent parameters have been extracted from melting experiments of short duplexes of varying sequences and lengths [10,54,55,56] for both DNA and RNA and are accessible in the Mfold server [11]. Single-molecule techniques allow for much more accurate free-energy measurements with respect to bulk experiments. However, to derive the 10 (8 if circular symmetry is considered) NNBP parameters from unzipping experiments, it is fundamental to have a theoretical model of the unzipping process to predict the experimental FDC.

### 3.2. Computation of the System Free-Energy

In unzipping experiments at controlled position, the trap–pipette distance, $x_{\mathrm{tot}}$, is steadily increased (unfolding) or decreased (refolding) by moving the position of the optical trap. As the optical trap moves, the force *f* applied on the molecular construct changes and the number of open bases *n* varies accordingly. The total trap–pipette distance equals the sum of different contributions [57] (see Figure 3C), that is:

(3) $$x_{\mathrm{tot}}\left(f,n\right)=x_{b}\left(f\right)+2x_{h}\left(f\right)+2x_{ss}\left(f,n\right)+x_{d}\left(f\right)$$

where $x_{b}\left(f\right)$ is the displacement of the bead in relation to the center of the optical trap, $x_{h}\left(f\right)$ is the extension of the double-stranded handles, $x_{ss}\left(f,n\right)$ is the extension of the single-stranded (unfolded) molecule containing *n* bases, and $x_{d}\left(f\right)$ accounts for the extension of the diameter of the NA helix *d* (typically d=2 nm for DNA and RNA hairpins [58]) projected along the force axis [59]. Notice that the latter term is zero when the hairpin is fully unzipped. The bead in the optical trap is modeled as a linear spring of stiffness *k*:

(4) $$f\left(x_{b}\right)=kx_{b}.$$

The double-stranded handles and the single-stranded NA are modeled as elastic polymers. Typically, these terms are described by the inextensible or extensible Worm-Like Chain (WLC) model [60]. In the former case:

(5) $$f\left(x\right)=\frac{k_{B}T}{4l_{p}}\left[{\left(1-\frac{x}{nl_{d}}\right)}^{-2}-1+4\frac{x}{nl_{d}}\right]\;$$

where $k_{B}$ is the Boltzmann constant, *T* is the temperature, $l_{p}$ is the persistence length, and $l_{d}$ is the interphosphate distance. Finally, the extension upon orienting the double helix is modeled as a dipole of length equal to the helix diameter, *d*, that aligns along the force axis:

(6) $$x_{d}\left(f\right)=d\left[\coth\left(\frac{fd}{k_{B}T}\right)-\frac{k_{B}T}{fd}\right].$$

Notice that while $x_{b}\left(f\right)$ and $x_{d}\left(f\right)$ are directly obtained from Equations (4) and (6), respectively, $x_{ss}\left(f,n\right)$ requires inverting Equation (5) [57].

For a given $x_{\mathrm{tot}}$ and *n*, the total free-energy of the NA hairpin is given by these elastic terms plus the hybridization free-energy in Equation (1) so that:

(7) $$\Delta G_{\mathrm{tot}}\left(x_{\mathrm{tot}},n\right)=\Delta G_{b}\left(x_{b}\right)+2\Delta G_{h}\left(x_{h}\right)+2\Delta G_{ss}\left(x_{ss},n\right)+\Delta G_{d}\left(x_{d}\right)+\Delta G_{0}\left(N-n\right)$$

where *N* is the total number of base-pairs in the sequence and the distance constraint Equation (3). The elastic free-energy contributions are obtained by computing the following integral:

(8) $$\Delta G_{el}\left(x\right)=\int_{0}^{x}f\left(x^{\prime }\right)dx^{\prime }$$

of Equations (4)–(6). Therefore, it is straightforward to obtain:

(9) $$\Delta G_{b}=\frac{1}{2}kx_{b}^{2}$$

and

(10) $$\Delta G_{\mathrm{WLC}}\left(x\right)=\frac{k_{B}T}{4l_{p}}\left[nl_{d}{\left(1-\frac{x}{nl_{d}}\right)}^{-1}-x+2\frac{x^{2}}{nl_{d}}\right]$$

from Equations (4) and (5), respectively. The computation of the free-energy needed to orient the dipole-like folded hairpin requires inverting Equation (6).

### 3.3. The Equilibrium FDC

Given the above model, it is possible to compute the equilibrium force of the system, $f_{eq}$, at each instant of the unzipping protocol. This ultimately allows us to obtain a theoretical prediction of the equilibrium FDC of the hairpin sequence. To do this, let us introduce the system partition function for a fixed $x_{\mathrm{tot}}$, which is defined as the sum over all the possible states (all the possible values of *n*) so that:

(11) $$Z\left(x_{\mathrm{tot}}\right)=\sum_{n=0}^{N}\exp\left(-\frac{\Delta G_{\mathrm{tot}}\left(x_{\mathrm{tot}},n\right)}{k_{B}T}\right)\;$$

where *N* is the total number of base-pairs of the sequence. By recalling that $\Delta G=-k_{B}T\ln Z$, the equilibrium force is then:

(12) $$f_{\mathrm{eq}}\left(x_{\mathrm{tot}}\right)=-k_{B}T\frac{\partial \ln Z(x_{\mathrm{tot}})}{\partial x_{\mathrm{tot}}}.$$

Computing Equation (11) requires solving the transcendental Equation (3) (that can be performed numerically) with respect to *f* and then computing Equation (7) for all n∈[0,N]. The value $n^{*}$ minimizing the equilibrium free-energy $\Delta G_{\mathrm{eq}}=\Delta G_{\mathrm{tot}}\left(x_{\mathrm{tot}},n^{*}\left(x_{\mathrm{tot}}\right)\right)$ gives the most probable number of open base-pairs at a given $x_{\mathrm{tot}}$. Eventually, the computation of the equilibrium force in Equation (12) gives a theoretical prediction for the unzipping curve of a given sequence (see Figure 3D). Notice that the total energy in Equation (7) is given by the balance between two independent contributions: the elastic terms and the hybridization energy. The latter term grows linearly with *n* (and does not depend by $x_{\mathrm{tot}}$) while the elastic one is a non-linear function of $\left(x_{\mathrm{tot}},n\right)$. This means that a variation of $x_{\mathrm{tot}}$ does not necessarily imply a variation of $n^{*}$. As a result, the equilibrium energy $\Delta G_{\mathrm{eq}}$ is a rough function of $x_{\mathrm{tot}}$ exhibiting a sequence of steps (Figure 3D, inset) as $n^{*}$ changes upon releasing groups of $\Delta n^{*}$ bases. Each one of these jumps corresponds to a rip along the equilibrium FDC (Figure 3D).

## 4. Derivation of the NNBP Free-Energies

Unzipping data for long NA hairpins have been used to derive the formation free energies of the 10 NNBP motifs in DNA [33,34] and RNA [35]. To do this, we developed a Monte Carlo optimization algorithm that uses both the experimental data and the theoretical FDC prediction described in Section 3 [33]. By starting with an initial guess of the 10 independent parameters (or 8 if circular symmetry is applied), at each step of the optimization, a random increment in the energies is proposed and a prediction of the FDC is generated. The error made in approximating the experimental curve with the theoretical one, *E*, drives a Metropolis algorithm: a change in the energy parameters is accepted if the error difference with respect to the previous step is negative (ΔE<0). Otherwise (ΔE>0), the proposal is accepted if exp(ΔE/T)<r, with *r* being a random number uniformly distributed r∈U(0,1). The algorithm continues until convergence is achieved, i.e., until ΔE is smaller than a given threshold. Let us note that because of the high number of parameters, only experimental data from the unzipping of long molecules (a few kbp) allow for an accurate estimation of the NNBP energies. In fact, the algorithm relies on the saw-tooth pattern characteristic of the sequence to accept or reject an energies proposal: the longer the sequence is, the more accurate the values of the NNBP energies are.

**Figure 4:**
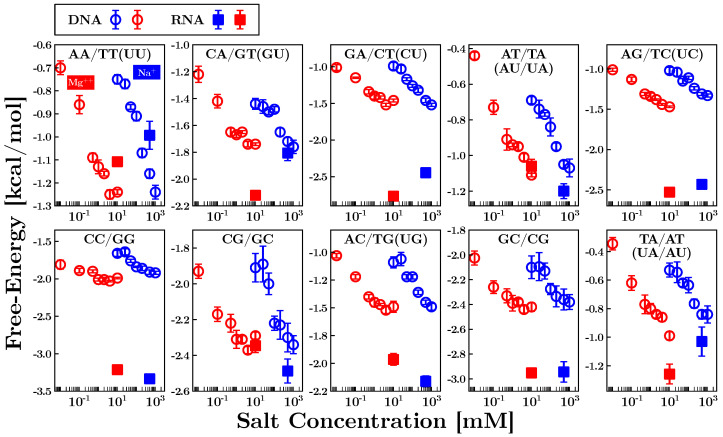
Experimentally measured NNBP free-energies of DNA (circles) and RNA (squares) at different salt concentrations of sodium (blue) and magnesium (red). The salt dependence of the NNBP energies in DNA has been studied in the ranges [10, 1000] mM NaCl and [0.01, 10] mM MgCl ${}_{2}$ [34]. In RNA, the energies have been measured at 500 mM NaCl and 10 mM MgCl ${}_{2}$ [35]. The energy of the last two motifs, GC/CG and TA/AT (UA/AU for RNA), has been computed by using the circular symmetry relations (see Equations (2)).

In Figure 4, we show the results obtained for the eight independent NNBP free-energies of DNA (circles) and RNA (squares) at different concentrations of sodium (blue) and magnesium (red). In particular, the DNA energies have been measured in a salt range from 10 mM to 1 M for Na ${}^{+}$ and from 0.01 mM to 10 mM for Mg ${}^{++}$, while the RNA energies have been measured at 500 mM Na ${}^{+}$ and 10 mM Mg ${}^{++}$. The last two parameters, GC/CG and TA/AT (UA/AU for RNA), were obtained by applying the circular symmetry relations. It is apparent that the (negative) energy parameters are lower for RNA than for DNA. Notice that this difference is more marked for motifs containing at least one purine (A, G), i.e., where stacking interactions are stronger, explaining why RNA tends to fold into more compact structures than DNA does [61].

Moreover, the NNBP RNA energies have been used to test the validity of the 1/100 salt equivalence rule of thumb. The rule states that the RNA energy parameters at a given divalent salt concentration equal that at a 100-fold monovalent salt concentration, i.e., $\Delta g_{i}\left[{\mathrm{Div}}^{++}\right]=\Delta g_{i}{\left[{\mathrm{Mon}}^{+}\right]}_{\mathrm{eq}}$, where ${\left[{\mathrm{Mon}}^{+}\right]}_{\mathrm{eq}}=100\times \left[{\mathrm{Div}}^{++}\right]$. Our results proved that 10 mM ${\mathrm{Mg}}^{++}$ corresponds to 800 mM ${\mathrm{Na}}^{+}$ (approximately a 1/80 equivalence), which is compatible with the 1/100 rule within the experimental errors [35]. Although this result has been tested in a single-salt condition, its validity extends to the dilute salt regime where cooperative salt effects are negligible ($[{\mathrm{Mg}}^{++}]<0.05\;M$) and competition effects with sodium are weak ($R=\sqrt{\left[{\mathrm{Mg}}^{++}\right]}/\left[{\mathrm{Na}}^{+}\right]>0.22M^{-1/2}$) [62,63]. The 1/100 salt equivalence rule of thumb has been disputed on the basis of experimental data obtained in bulk experiments using atomic emission spectroscopy in buffer-equilibrated samples [64]. Although this technique is capable of determining the fraction of cations that are dissociated and bound to the RNA, it does not provide a direct measurement of the free energies.

## 5. Out-of-Equilibrium Processes and Kinetics Effects

**Figure 5:**
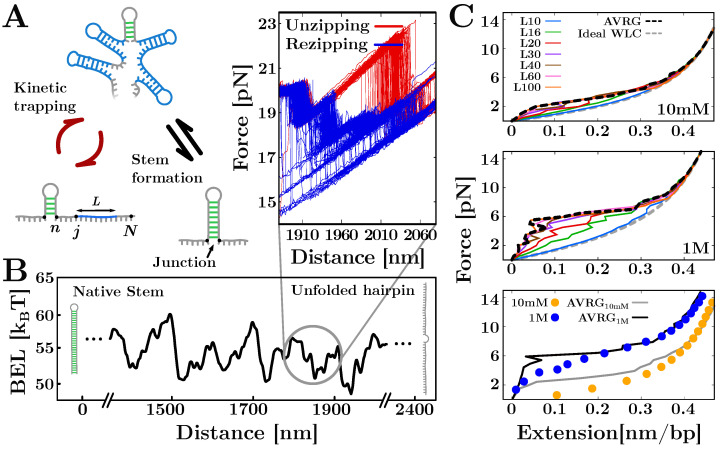
Stem-loops’ formation along the single-stranded sequence. (**A**) Kinetic trapping during unfolding and refolding processes allows for the formation of stem-loops of different lengths folding along the unpaired strands of the molecule. As the state of highest stability is always the native state, the stem eventually forms. (**B**) Loop-BEL profile as a function of the trap–pipette distance. The zoom shows the FDC region corresponding to the circled loop-BEL. The minima of the landscape correlates with the measured hysteresis. (**C**) Extension of ssDNA predicted by the BEL model for different *L*-segments (solid lines) and over all *L* (dashed black lines) at 10 mM and 10 M NaCl (top and middle panels). The comparison between these predictions (solid lines) and experiments (solid dots) shows that the model reproduces the observed formation of non-specific secondary structure (bottom panel). The deviation form this behavior at low force (f<6 pN) is due to the approximation made in computing the model.

To characterize the irreversibility observed along the unzipping FDCs for RNA (see Figure 2), we introduced a many-valley barrier energy landscape (BEL) that accounts for the off-pathway competing stem-loop structures that can form along each single strand of the hairpin [35]. Upon unfolding (refolding) the hairpin, multiple stem-loops of varying lengths *L* can form along the single strands of the hairpin, i.e., in the vicinity of the hybridization junction separating the stem from the ssNA (Figure 5A, bottom right). Stem-loops slow down the hybridization of the native stem by stabilizing off-pathway conformations that kinetically trap the multiple apparent intermediates observed along the FDC (Figure 5B, grey circle snapshot). These complex structures are transiently stable; therefore, the stem-loops eventually unfold, and the native stem can form (Figure 5A).

We model the loop-BEL as follows (see Ref. [35] for details). Let us consider the set of all possible segments of *L* bases along a single-stranded sequence of *N* bases, $S_{L}=\left\{\left[b_{i},b_{i+L}\right];\;1\leq i\leq N-L\right\}$, where $b_{i}$ and $b_{i+L}$ are the initial and the final bases of the segment, respectively. For a given *L*-segment, $\left[b_{i},b_{i+L}\right]$, there are several competing folds, most of them stabilized by a few Watson–Crick base-pairs plus one or more loops of varying sizes (mostly from three to eight bases). By using the DINAmelt web application [65,66], we found the minimum free energy fold for a given segment. This gives the set of lowest free energy folds, $\left\{\epsilon_{L,i}^{0}\right\}$ for all *L*-segments in $S_{L}$ at standard conditions. To construct the loop-BEL, one should consider all possible excitations (i.e., higher energy states) formed by multiple stem-loops folding along the two ssDNA strands at both sides of the junction. In principle, any number of stem-loops can form at arbitrary positions along the two strands. As counting all possible excitations is a daunting task, in the simplest approach, one can simplify the treatment by considering two stem-loops (one per strand) that are located at arbitrary and independent positions. However, stem-loops that are located far away from the junction, even if energetically favorable, cannot interfere with the unzipping–rezipping of the hairpin, a reaction taking place precisely at the junction. For a given stem-loop at a distance *j* from the junction and a given force *f*, we introduce an energy penalty equal to the mechanical work at constant *f* needed to bring that stem-loop from distance j+L to the junction, $\int_{0}^{f}x_{j+L}\left(f^{\prime }\right)df^{\prime }$ (Figure 5A, bottom left).

Given *L*, we define the loop-BEL at force *f* and junction position *n* as:

(13) $$\Delta G_{L}\left(n,f\right)=-k_{B}T\log\sum_{j_{1},j_{2}=0}^{N-n}\exp\left(-\frac{\Delta g_{L}^{\left(1\right)}\left(j_{1},f\right)+\Delta g_{L}^{\left(2\right)}\left(j_{2},f\right)}{k_{B}T}\right),$$

where $\Delta g_{L}^{\left(1,2\right)}\left(j,f\right)$ is the free-energy of forming a single stem-loop in strand (1,2) of length *L* plus the work at force *f* to bring it from position *j* to the junction located at position *n*. $\Delta g_{L}^{\left(1,2\right)}\left(j,f\right)$ is then given by:

(14) $$\Delta g_{L}^{\left(1,2\right)}\left(j,f\right)=\epsilon_{L,j}^{0(1,2)}+\int_{0}^{f}x_{L+j}\left(f^{\prime }\right)df^{\prime }\;.$$

Here, $\epsilon_{L,j}^{0(1,2)}$ is the (negative) free energy of formation at zero force of the stem-loop $\left[b_{j},b_{j+L}\right]$ in strand (1,2). The integral term accounts for the free energy cost at force *f* to bring the base of the stem-loop located at the farthest end, j+L bases away from the junction, towards the junction. As previously said, this term penalizes stem-loops that are formed far away from the junction because they cannot kinetically trap the native RNA hairpin. The elastic response term $x_{j+L}\left(f\right)$ has been modeled with the WLC in Equation (5).

We found that the minima of the loop-BEL (Figure 5B) directly correlate with the amount of hysteresis observed along the FDC [35]. This correlation is maximum for stem-loops of length L∼20 bases, indicating that *L*-segments of this size are the most likely to fold. Moreover, the analysis of the hairpin sequence showed that the regions of large irreversibility contain a high fraction of stacked (A, U) bases that are prone to Watson–Crick pairing within the same strand. On the contrary, regions of low hysteresis are characterized by lower stacking and intra-strand pairing interactions. The formation of stem-loops structures may contribute to explaining the broad phenomenology of heterogeneous RNA folding, from misfolding and multiplicity of native states to the formation of complex tertiary structures. In particular, the BEL model can be extended to the formation of non-specific secondary structures observed in pulling experiments of ssDNA and ssRNA. Although this phenomenon has not been investigated in RNA, it has been extensively studied in DNA [28,30,67,68,69]: upon stretching, the ssDNA’s elastic response deviates from the expected ideal behavior of a polymeric chain (described for example with the WLC) forming a shoulder below f∼ 10–12 pN (see Figure 5C, bottom).

To model this phenomenon, let us consider an ssDNA of *N* bases of a random sequence, which, at difference with the previous case, cannot form a native hairpin (i.e., there is no hybridization junction). We consider the set of all possible excitations consisting of multiple stem-loops of a given length *L* along the sequence. The free energy of such an *L*-set of excitations equals:

(15) $$\Delta G_{L}\left(f\right)=-k_{B}T\log\sum_{k=0}^{K}\exp\left(-\frac{\Delta g_{L}\left(k,f\right)}{k_{B}T}\right)\;,$$

where $\Delta g_{L}\left(k,f\right)$ is the total free-energy contribution of k≥0 stem-loops and K=⌊N/L⌋ is the maximum number of stem-loops that can form along the single strand. This is given by:

(16) $$\Delta g_{L}\left(k,f\right)=E_{L}\left(k\right)+\left[\left(N-kL\right)\Delta G_{ss}^{1}\left(f\right)+k\Delta G_{d}\left(f\right)\right]\;.$$

The term $E_{L}\left(k\right)$ accounts for the most energetically stable configuration of *k* stem-loops randomly positioned along the sequence. The term $\left(N-kL\right)\Delta G_{ss}^{1}\left(f\right)$ is the energy gain upon stretching the free N−kL bases at force *f* corrected by the (smaller) energy contribution, $k\Delta G_{d}\left(f\right)$, of orienting *k* stem-loops along the force axis. Notice that $\left(N-kL\right)\Delta G_{ss}^{1}\left(f\right)$ is an extensive quantity, equal to the number of monomers, N−kL, times the energy cost to stretch a single monomer, $\Delta G_{ss}^{1}\left(f\right)=-\int_{0}^{f}x_{ss}^{1}\left(f^{\prime }\right)df^{\prime }$, where $x_{ss}^{1}\left(f\right)$ has been modeled according to the WLC in Equation (5). The same consideration holds for the dipole contribution of *k* stem-loops, $\Delta G_{d}\left(f\right)=-\int_{0}^{f}x_{d}\left(f^{\prime }\right)df^{\prime }$, where $x_{d}\left(f\right)$ is modeled according to Equation (6).

An exact computation of $E_{L}\left(k\right)$ in Equation (16) requires considering non-overlapping stem-loops: if a stem-loop of length *L* forms at position *n* along the sequence, the next stem-loop can only form outside the interval [n−L:n+L]. This is an unaffordable mathematical task that we simplified by considering overlapping stem-loops in a mean-field approximation. In this approximation, $E_{L}\left(k\right)$ is taken as the typical total energy of *k* stem-loops randomly chosen over the ensemble of $C_{k}$ different realizations, without imposing any constraints on these loops (i.e., they can be overlapping or non-overlapping). In contrast, the stretching contribution $\left(N-kL\right)\Delta G_{ss}^{1}\left(f\right)+k\Delta G_{d}\left(f\right)$ is taken independent of the *k* stem-loops realization. Therefore, we have:

(17) $$E_{L}\left(k\right)\approx \min_{C_{k}}\left\{\sum_{k}\epsilon_{L,k}^{0}\right\}-k_{B}T\log\left(C_{k}\right)\;,$$

where, for the typical energy of *k* stem-loops ($\epsilon_{L,k}^{0}<0,\forall k,L$), we took the most stable configuration (i.e., the one of lowest energy) within the ensemble $C_{k}$. For large *N*, the total number of configurations in $C_{k}$ is enormous, so we restricted the sampling to a few hundreds of configurations (typically 500). The second term in the rhs of Equation (17) is an entropic contribution stabilizing the formation of stem-loops. The total number of configurations is given by the binomial coefficient, Ck=Kk, or the number of ways *k* objects (stem-loops) can be arranged into K=⌊N/L⌋ different positions.

From Equations (15) and (16), we can compute the average ssDNA extension for a given *L*, which is defined as:

(18) $$x_{ss,L}\left(f\right)=-\frac{\partial \Delta G_{L}\left(f\right)}{\partial f}=\frac{1}{Z_{L}}\sum_{k=0}^{K}x_{L}\left(k,f\right)\exp\left(-\frac{\Delta g_{L}\left(k,f\right)}{k_{B}T}\right)\;,$$

where $Z_{L}$ is the system’s partition function for a given *L* (c.f. Equation (15)):

(19) $$Z_{L}=\exp\left(-\frac{\Delta G_{L}\left(f\right)}{k_{B}T}\right)=\sum_{k=0}^{K}\exp\left(-\frac{\Delta g_{L}\left(k,f\right)}{k_{B}T}\right)$$

and $x_{L}\left(k,f\right)$ is the ssDNA extension when *k* stem-loops are formed:

(20) $$x_{L}\left(k,f\right)=\left(N-kL\right)x_{ss}^{1}\left(f\right)+kx_{d}\left(f\right)\;.$$

Finally, the thermodynamic free-energy and the ssDNA extension averaged over all *L*-segments are computed as:

(21) $$\Delta G\left(f\right)=-k_{B}T\log\sum_{L}\exp\left(-\frac{\Delta G_{L}\left(f\right)}{k_{B}T}\right)\;.$$

and

(22) $$x_{ss}\left(f\right)=\frac{1}{Z}\sum_{L}x_{ss,L}\left(f\right)\exp\left(-\frac{\Delta G_{L}\left(f\right)}{k_{B}T}\right)\;,$$

where $Z=\sum_{L}Z_{L}$ is the system’s partition function.

In Figure 5C we show the ssDNA extension predicted by the BEL model for a random DNA sequence of N=2027 bases at 10 mM (top panel) and 1 M (middle panel) NaCl salt concentrations. These results are compared with experimental data from pulling experiments of a long DNA hairpin in the same salt conditions (bottom panel). The expected ssDNA elastic response for L∈[10,100] computed with Equation (18) (solid lines) is shown along with $x_{ss}\left(f\right)$ in Equation (22) (dashed black line). It is apparent that the BEL model reproduces the deviation from the ideal WLC model (dashed gray line) experimentally observed below ∼10 pN. This behavior results from the competition between the stem-loops of different sizes: the lower the force, the larger the contribution to Equation (22) by larger stem-loops. Therefore, as the ssDNA approaches the random coil state (f=0 pN), the energetic gain to stretch large *L*-segments in Equation (16) tends to zero, while $E_{L}\left(k\right)$ remains constant, favoring the formation of large stem-loop structures. Remarkably, as the force approaches the dsDNA unzipping force (f≈15 pN), stem-loops of length L∼ 30–40 bases become the most likely folds. This number is not far from what has been reported for RNA, where L∼20 (see above and Ref. [35]). At higher forces, the elastic response collapses to the WLC as experimentally observed. Let us notice that at both 10 mM and 1 M NaCl, the predicted extension differs from the experimental data (solid dots in Figure 5C, bottom) when f<6 pN. This is particularly evident at 10 mM NaCl as our model indicates that stem-loops still form at low force while no secondary structure is observed in the pulling trajectories. This and other potential inconsistencies come from the crude approximations made in Equation (17). In fact, this approximation only holds when k≪K, i.e., when the typical distance between consecutive stem-loops is much larger than *L*, so that the overlapping is negligible. Despite the simplicity of the mean-field approximation, the BEL model is useful to study the complex behaviors observed in NA. A more rigorous analytical treatment may lead to a deeper understanding of heterogeneous folding in NA.

## 6. Discussion

We discussed recent advances in single-molecule pulling experiments with laser optical tweezers (LOT) to investigate NAs’ thermodynamics. Many of the results that we have presented cannot be obtained with bulk methods, which, by averaging over populations of molecules, do not permit sampling rare NAs folds such as misfolded configurations and intermediates. In addition to accurate experimental measurements, it is also key to have a reliable thermodynamic model for the unzipping reaction. In Section 3, we described the nearest-neighbor (NN) model that reproduces the experimental force–distance curves (FDCs). To date, the NN model provides the best theoretical description of the unzipping experiments. Conversely, unzipping experiments provide an elegant verification of the NN model. Our experimental-theoretical approach ultimately allows for predicting the unzipping FDC and the extraction of the nearest-neighbor base-pair (NNBP) energy parameters with 0.1 kcal/mol accuracy.

In Section 4, we summarized the NNBP energy parameters for DNA [34] and RNA [35]. Remarkably, the results are in agreement with the literature based on bulk assays [9,10]. The difference observed between the DNA and RNA energy parameters, where energies are lower for RNA, explains the higher stability of dsRNA duplexes in melting and unzipping experiments. It also explains the slower kinetics in RNA folding, where heterogeneous structures are often observed. This is confirmed by the multiple intermediates visible in the RNA FDCs that are not observed in DNA (Figure 2. Even though the irreversibility in RNA have been observed before in pulling experiments of short duplexes [70], a characterization of this general phenomenon in the unzipping of long RNA hairpins has been fundamental to measure the RNA NNBP free-energies. We do not exclude the possibility that similar intermediates also occur in DNA folding; however, this would require different experimental conditions, such as lower temperatures [5,71].

In Section 5, we have shown that the formation of stem-loops along the single strands explains the kinetic phenomena observed in unzipping experiments. To model this mechanism, we used the free-energy landscape formalism, useful to understand protein and NA folding into complex tertiary structures [5,72]. On the one hand, the barrier energy landscape (BEL) model introduced in Ref. [35] and defined in Equation (13) correlates with the hysteresis measured along the RNA FDCs. This shows that stem-loops are off-pathway structures that kinetically trap the RNA hairpin, slowing down the formation of the stem. On the other hand, the BEL model allows for predicting the formation of non-specific secondary structure in ssDNA pulling experiments [30]. This feature appears to be caused by the competition between multiple stem-loop folds of different sizes that make the extension to deviate from the ideal WLC behavior. Stem-loops formation appears as a general mechanism driving the folding and refolding processes of RNA hairpins as well as the elastic response of ssDNA sequences, and it may help understanding the broad phenomenology showed by NAs. However, the predictions obtained by the present model are limited by crude approximations introduced to simplify the enormous complexity of a complete stem-loops modeling. Firstly, in the computation of the stem-loops free-energy (Equations (16) and (17)), we disregarded overlapping effects between consecutive L-segments. This implies that partially overlapping L-segments can simultaneously fold into stem-loops. Moreover, we neglected the cooperativity effects, which favor the nucleation of contiguous stem-loops in an avalanche fashion reminiscent of phase transitions. Ultimately, a full BEL modeling would require allowing for the simultaneous formation of stem-loops of different lengths *L*, whereas the current model accounts for this effect through a mean-field approximation (Equations (21) and (22)). Although a comprehensive modeling of stem-loops formation is lacking, the phenomenon might explain many features of heterogeneous NA folding. The development of a more accurate description of the BEL model accounting for the complex phenomenology discussed above is left for future work.

## Data Availability

The results shown in Figure 4 and Figure 5 have been published in [34,35], respectively. All the other results are original and will be shared upon request.
